# NEONATAL PAIN: CHARACTERIZATION OF THE PHYSIOTHERAPIST’S PERCEPTION IN THE NEONATAL INTENSIVE CARE UNIT

**DOI:** 10.1590/1984-0462/2020/38/2018178

**Published:** 2019-11-25

**Authors:** Isabelle Leandro Gimenez, Vanessa da Silva Neves Moreira Arakaki, Raquel Miranda Correa, Rosana Silva dos Santos, Rodrigo Tosta Peres, Clemax Couto Sant’Anna, Halina Cidrini Ferreira

**Affiliations:** a Universidade Federal do Rio de Janeiro, Rio de Janeiro, RJ, Brazil.; b Centro Federal de Educação Tecnológica Celso Suckow da Fonseca do Rio de Janeiro, Rio de Janeiro, RJ, Brazil.

**Keywords:** Pain, Pain measurement, infant premature, physiotherapy, neonatal intensive care unit, Dor, Mensuração da dor, Recém-nascido prematuro, Fisioterapia, Terapia intensiva neonatal

## Abstract

**Objective:**

To describe the perception of physiotherapists in neonatal units regarding pain, the use of measurement scales and strategies that minimize pain.

**Methods:**

Interviews were conducted with physiotherapists in hospitals with neonatal units between 2013 and 2015 in Rio de Janeiro. The questions concerned the knowledge of the feeling of pain, from its recognition to its care or treatment. The description of the results was done by comparing public and private hospitals (Fisher’’s Exact exact Testtest), considering p<0.05 as significant.

**Results:**

27 hospitals were visited. All the professionals interviewed (n=27) stated that the newborns feel pain, with facial expression being the most cited and known sign for pain. 26% of physiotherapists believe that newborns experience pain at the same magnitude as adults. Among the scales, the Neonatal Infant Pain Scale (NIPS) was the most well known, but only 37% of the units had routine pain assessment protocols. IV cannulation and blood collection were the most mentioned procedures as a cause of pain and there was no difference between public and private hospitals.

**Conclusions:**

There is a gap in the knowledge about neonatal pain and how to evaluate it among the participating physiotherapists, with no systematization of care routines involving this assessment.

## RESUMO

**Objetivo:**

Descrever a percepção dos fisioterapeutas de unidades neonatais sobre a dor, a utilização de escalas de mensuração e estratégias que a minimizem.

**Métodos:**

Entrevistas foram realizadas com chefes ou rotinas de fisioterapia em hospitais com unidades neonatais entre 2013 e 2015, no Rio de Janeiro. As perguntas versaram sobre o conhecimento da sensação dolorosa, desde seu reconhecimento até seu cuidado ou tratamento. Foi realizada a descrição dos resultados, comparando-se os dados dos hospitais públicos com os privados (teste exato de Fisher), considerando-se p<0,05 como significante.

**Resultados:**

Vinte e sete hospitais foram visitados. Todos os profissionais entrevistados (n=27) afirmaram que os recém-nascidos sentem dor, sendo a expressão facial o sinal de dor mais conhecido. Do total de fisioterapeutas entrevistados, 26% acreditam que os neonatos sentem dor na mesma magnitude que o adulto. Entre as escalas, a Neonatal Infant Pain Scale (NIPS) era a mais conhecida, e apenas 37% das unidades possuíam protocolos de avaliação da dor na rotina. As coletas e as punções foram os procedimentos mais mencionados como causa de dor, e não houve diferença entre os hospitais públicos e privados.

**Conclusões:**

Constatou-se uma lacuna no conhecimento sobre dor neonatal e como avaliá-la entre os fisioterapeutas participantes, com ausência de sistematização de rotinas assistenciais que envolvam essa aferição.

## INTRODUCTION

Pain can be defined as an unpleasant physical and emotional experience associated with a real, potential or described tissue injury which is always subjective.^1^ However, this concept cannot be applied literally to newborns (NBs) due to lack of their ability to verbalize and the absence of previous painful experiences that would enable the comparison and description of the feeling of pain.^2^ Nevertheless, it is important to consider that a pre-verbal individual feels pain,^1^ ^,^ ^3^ ^,^ ^4^ ^,^ ^5^ and that measures against painful stimulus should be put into practise^1^ since chronic exposure can lead to future learning, cognition, and emotional impairments, as well as behavioral changes and impairment of newborn growth.^6^ ^,^ ^7^ ^,^ ^8^

Since 1989, pain has been highlighted as the fifth vital sign^9^ and in NBs it is measured by sometimes subjective instruments, without one that can be classified as the gold standard.^8^ Considering that the newborn cannot verbalize, monitoring pain in this population is a challenge and consists of observing physiological and behavioral alterations.^6^ ^,^ ^10^ ^,^ ^11^

Newborns admitted to neonatal units should be cared for by a multidisciplinary team. Physiotherapists are part of this team and perform various manoevers during their routine of care. Therefore, the professional should be encourgaed to minimize any unpleasant feelings in the newborn in order to ensure the excellence of care. Reports on the presence of pain in newborns and the various strategies for its reduction have been growing and, although still low in number, have been able to arouse attention and the search for the introduction of this type of assessment in many hospitals, however probably without systematization of care routines.^12^ ^,^ ^13^ ^,^ ^14^ ^,^ ^15^

The objective of this study was to describe the perception of physiotherapists of neonatal intensive care units in Rio de Janeiro regarding pain, the use of scales and instruments that measure it and strategies that minimize it during routine procedures.

## METHOD

A field research with a cross-sectional, descriptive appraoch, approved by the Ethics and Research Committee of the Maternity School of the Universidade Federal do Rio de Janeiro, under CAAE number: 25211913.9.0000.5275.

A listt of all hospitals in a large Brazilian city that offered this type of assistance between 2013 and 2015 was made through consultations with associations and scientific societies related to the area, in addition to searching and confirming on the internet. In addition, telephone contacts and site visits for confirmation and invitations to participate were made (informed consent). Hospitals that offered neonatal intensive care and whose physiotherapist manager or staff physiotherapists agreed with the study, were included. Mixed units with pediatric care were excluded from the study.

Interviews were conducted with a manager or a physiotherapist who worked in the service and asked questions about knowledge regarding the pain process, from its recognition to its proper care / treatment in newborns. The information provided by the physiotherapists referred to themselves and the team they led. The form used by the group of researchers was completed by the researchers during the visits and contained 18 easy-to-understand open or closed questions that allowed to profile the professionals and understand the studied subject (form available with corresponding author). The distribution of shifts, type of department and the frequency of meetings and discussions were collected according to Arakaki et al.^16^ The collected data were stored in an Excel spreadsheet and the analysis was performed by summarizing, organizing, and describing the set of results in descriptive statistics, in absolute numbers and percentages. Additionally, the number of citations in each reality was compared to the total in each(public / private) by Fisher’s Exact Test (RStudio, with R programming language), considering p<0.05 as significant.

## RESULTS

After 34 visits to hospitals, 27 were included (17 from public institutions and 10 from private institutions). The included hospitals are geographically distributed in the city of Rio de Janeiro, with a higher concentration in the port, central and southern areas of the municipality. Areas further away from the city have fewer neonatal units, despite being highly populated areas. ^16^ The exclusions were due to the absence of the physiotherapist in one of the hospitals and refusal to participate in six institutions.

The profile of hospital physiotherapy managers / routine physiotherapists was 100% of professionals with more than two years of professional experience and 16 managers / routine physiotherapists are neonatal intensive care specialists (62.5% in public hospitals and 37.5% in private hospitals). Regarding experience in neonatology, it was observed that 22 managers / routine physiotherapists had 5 years or more of experience (55% in public hospitals and 45% in private hospitals) and only 5 (all from public hospitals) had between 2 and 4 years of experience in neonatology.

Regarding the distribution of physiotherapy shifts, five hospitals provide care between six and nine hours a day (90% public and 10% private), 11 hospitals work with 12-hour shifts (45.4% public and 54.5% private) and eight hospitals include 24-hour shifts (62.5% public and 37.5% private). The other hospitals only perform physical therapy visits, in which the physiotherapist does not remain on duty, and only responds to requests. Most of the visited units v(65%) have exclusive physiotherapy service to the department, but in the remaining institutions, the professional who covers the neonatal ICU also covers other departments. Meetings aimed to update professionals and have scientific discussions, take place in only eight (30%) of the visited institutions (6 in public hospitals and 2 in private hospitals).

All the interviewed professionals (n = 27) state that newborns feel pain and, for 26% of the managers/ routine physiotherapists, newborns feel pain in the same way as adults, while 74%, state that the feeling would be different. All professionals reported being able to recognize pain through the following signs: facial expression (100%), heart rate change (59%), respiratory rate change (52%), SpO_2_ change (48%) and skin coloration (44%). In addition, the following were mentioned: crying, limb movement and irritability as forms of pain recognition. The details of the pain recognition signs cited by the interviewed professionals can be seen in table 1. No significant differences were found between the citations according to the public / private reality.

**Table 1:** Most frequent signs of pain in newborns according to the physiotherapists interviewed in both types of units (public and private).

Signs of pain expression	Number of citations	Public hospitals	Private Hospitals	p- value*
Facial expression	27	17	10	1
Alteration in Heart rate	16	10	6	1
Alteration in respiratory rate	14	8	6	0.69
Alteration in oxygen saturation	13	8	5	1
Skin color	12	9	3	0.42
Cry	5	4	1	0.62
Irritability	3	2	1	1
Postural reflexes	1	1	0	1

*Comparison between the number citations in relation to the total in each of the realities (public / private) by Fisher’s Exact Test. There was no evidence of difference between the proportions.

All professionals interviewed considered it important to institute some strategy for the prevention and treatment of neonatal pain. The reasons mentioned for using such strategies were diverse, without a single conception, as reported below: “because pain changes the whole system of the child”, “to give comfort to the child, which will interfere with their general clinical status ”,“ pain interferes with the child’s response to treatment ”,“ to prevent future damage and intracranial bleeding ”,“ to decrease side effects ”,“ ´for better evolution ”,“ pain increases length of stay “aiming for homeostasis”, “pain worsens the general condition and hemodynamically destabilizes”, “for humanization”, “minimize respiratory and motor changes”, “no one likes to feel pain”, ” “Without pain, you can improve therapy in general.”

Regarding the neonatal pain assessment instruments, 16 (59%) of the interviewed physiotherapists knew at least one scale, 3 (11%) were unaware of any and 8 (30%) professionals did not answer the question. The most well known scale among professionals was the Neonatal Infant Pain Scale - NIPS, according to table 2. There was no statistically significant difference between the answers given in each of the realities (public / private).

**Table 2:** Pain scales known and cited by physiotherapists in both types of units - (public and private).

Scales	Number of citations	Public hospitals	Private hospitals	p- value*
NIPS	13	9	4	0.69
PIPP	1	1	0	1
NFCS	1	1	0	1
COMFORT	1	1	0	1
NI	8	5	3	1

NFCS: Neonatal Facial Coding System; NIPS: Neonatal Infant Pain Scale; PIPP: Premature Infant Pain Profile; NFCS: Neonatal Facial Coding System; COMFORT: Comfort scale; NI: not informed; * comparation between the number citations in relation to the total in each of the realities (public / private) by Fisher’s Exact Exact Test. There was no evidence of difference between the proportions.

It was observed that ten hospitals (37% - 4 private and 6 public) had protocols for pain assessment and / or service scale, while 17 (63% - 6 private and 11 public) did not have this routine. Most professionals did not know which scale was used in the unit, as it was not applied by physiotherapists.

**Figure 1:**
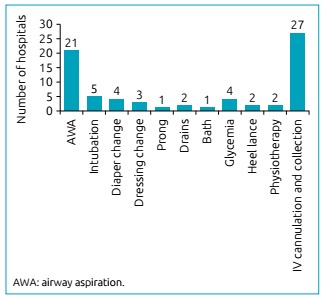
List of procedures cited by physiotherapists as potential causes of pain in neonates.

All professionals agreed that procedures performed in the neonatal ICU can potentially cause pain, especially IV cannulation and collection which were the most remembered procedures as a daily cause of pain, as shown in Figure 1.

Finally, all interviewed professionals, regardless of whether or not they measure neonatal pain in their routines, mentioned that they think it is important that general and global measures are taken to alleviate possible pain in the newborn, such as; bed positioning (93%); user embracement (89%); brightness reduction (81%); noise reduction 16 (59%); glucose use (55%); kangaroo method (52%); pharmacological analgesia (15%); network (7%); pacifier (7%) and, finally, warm hands before touching (4%). However, it is noteworthy that the verification of the effects of these measures is only measured by teams that use the scales as routine.

## DISCUSSION

This study highlights a gap in the knowledge of physiotherapists regarding neonatal pain, since evaluation and treatment occur intuitively, without systematization, routine organization or specific training on such care. There is limited data in Brazil regarding neonatal pain and the knowledge of the physiotherapist. In addition, the studies found do not individualize this professional category and therefore have a low number of physiotherapists, with emphasis on the team as a whole^17^ ^,^ ^18^ ^,^ ^19^.

It is possible to see that professionals from other areas caring for newborns in the neonatal ICU also do not have the specific knowledge regarding pain care and that there are discrepancies between clinical practice and perception regarding the presence or absence of painful stimulus^20^ ^,^ ^21^. Christoffel et al^.^ ^17^ demonstrated that, although most health professionals (66.3%) reported having obtained information on neonatal pain during their training, the most cited source of information for these professionals was managerial guidance and / or guidance from other health professionals, stating that knowledge about neonatal pain is gained without standardization.

Nursing is the professional category most commonly involved with the study of pain and its non-pharmacological treatment, with several publications on the subject.^17^ ^,^ ^22^ ^,^ ^23^ ^,^ ^24^ Given this reality, it is clear that even these professionals are still building consistent referential on standardization in NB pain care, as demonstrated by Soares et al^24^ in a study of 105 professionals.

These authors aimed to identify the knowledge of health professionals about pain management, assessment and treatment in a neonatal unit and found that, although claiming that scientific knowledge and practice should always be related, the means of evaluations did not confirm the discourse, confirming the importance of team training for bedside pain assessment.

As shown in Table 1, facial expression was cited by all professionals as a sign of pain. In fact, facial mimicry is widely used for pain perception in nonverbal individuals with good sensitivity and specificity.^25^ However, it has been shown that although facial movements are considered a good evaluation parameter, their absence does not mean that the newborn is not feeling pain. If there pain stimulus is repeated or if the newborn is premature, there may be less facial movement.^26^ Changes in heart and respiratory rate were well remembered and such statements are within what is expected to be observed with regard to pain in newborns.^9^ Ideally, the above mentioned physiological indicators should not be used in isolation, but combined for a more reliable measurement.^27^

Based on the need to combine physical and behavioral indicators, most newborn pain measurement scales use facial mimicry observation and physiological signs as tools for perception of unpleasant / painful stimuli. In this same line of thought, we questioned the knowledge of these scales and the best known among professionals was the NIPS, with a total of 13 (48%) answers, with 9 (69%) in public institutions and 4 (31 %) in private. There was no difference between the proportions of citations in each of the realities (public / private), demonstrating that there is no discrepancy between what is routinely done in relation to the management of newborn pain between different areas and locations. This fact has social relevance and shows that, regardless of the area and reality, routines related to pain need to be reviewed and included (Table 2). It is estimated that there are approximately 30 validated pain measurement scales for newborns, however, none of them are considered gold standard. With so many options in the literature, health professionals may have difficulties and doubts when choosing the most appropriate one.^28^

The reports of the physiotherapists and the citations mentioned as reasons for pain observation and scales presented a wide variety of answers, showing that there is a large knowledge gap that needs to be improved by discussions, training, and the systematization of routines. The same was also reported by Motta et al^23^ in a qualitative study with discourse analysis of health professionals from different areas. These authors concluded that it is important for multiprofessional teams to familiarize themselves with pain scales and pain management, using a different perspective based on training, detection and treatment. Prestes et al^20^ also emphasized the need to expand the training of health professionals regarding pain in the newborn, since the percentage of analgesia for procedures known to be painful in the four university neonatal ICUs studied was only 15% and that not all newborns in the immediate postoperative period after major surgery received analgesia.

Even though all the respondents were knowledgeable and members of the care teams, the study presented an important limitation that deserves to be noted: only the managers or routine physiotherapists of the units visited were heard due to the difficulty the researchers faced regarding the time and long distances to reach each professional of the teams. Ideally, all physiotherapists could have had a voice in these questions.

Knowledge about monitoring and the use of pain scales encourages the use of such instruments and broadens the perspective of professionals working in neonatal ICUs as well as their practices. It has been shown in nursing studies that, regardless of professional training, pain is perceived, however, the non-use of scales to standardize such measurement decreases the real clinical value of this measurement.^29^ ^,^ ^30^ It is important to emphasize the importance of educational interventions to improve the knowledge of the staff working in the neonatal ICU regarding pain management, which always leads to positive results for the care of newborns.^22^ ^,^ ^30^

Based on the results found, an important gap appeared in the knowledge regarding newborn pain and how to evaluate it among the physiotherapists working in these units, reaffirming the absence of systematization of care routines involving this measurement. Further studies are needed to broaden such findings as well as to propose care protocols involving the measurement of newborn pain
